# The Superconductivity in Bi-Doped BaFe_2_As_2_ Single Crystals

**DOI:** 10.3390/ma17040929

**Published:** 2024-02-17

**Authors:** Jiabin Si, Jianfa Zhao, Ying Liu, Ying Liu, Changqing Jin, Jibing Liu, Lingyi Xing

**Affiliations:** 1 College of Physics and Electronic Science, Hubei Normal University, Huangshi 435002, China; s18639161575@163.com (J.S.); 18715482798@163.com (Y.L.); liujb@hbnu.edu.cn (J.L.); 2 Beijing National Laboratory for Condensed Matter Physics, Institute of Physics, Chinese Academy of Sciences, Beijing 100190, China; jin@iphy.ac.cn; 3 Quantum Science Center of Guangdong-Hong Kong-Macao Greater Bay Area (Guangdong), Shenzhen 518045, China; liuying@quantumsc.cn

**Keywords:** BaFe_2_As_2_, iron-based superconductor, isovalent doping, negative pressure

## Abstract

We have successfully synthesized a series of Bi-doped BaFe_2_As_2_ high-quality single crystals for the first time. X-ray diffraction (XRD) patterns show an expansion of lattice parameter *c* with Bi doping, indicating a negative pressure effect. By investigating the resistivity, a Fermi liquid (FL) to non-Fermi liquid (NFL) crossover is observed from normal state to antiferromagnetic order state, accompanied by three superconducting transitions labeled as SC I, SC II and SC III, which are supposed to be induced by three superconducting realms with various Bi concentrations. Thus, we propose that the NFL behavior is closely related to the presence of superconductivity. The magnetic susceptibility measurements further speculate that the SC I and SC III phases should exhibit filamentary superconductivity while the SC II exhibits a possible bulk superconductivity of *T*_C_~7 K.

## 1. Introduction

Since the discovery of *T*_C_ = 26 K superconductivity in LaO_1−x_F_x_FeAs [1], iron-based superconductors (IBSC) have stimulated another round of interest in high-temperature superconductor research regarding cuprates [2]. Among them, the ‘122’ system *AE*Fe_2_As_2_ (*AE* = Ba, Sr and Ca) with a ThCr_2_Si_2_-type structure has been heavily studied because of its easily grown, large, suitable and high-quality single crystals [3,4,5]. As for the BaFe_2_As_2_ parent compound [3], it exhibits an anomaly transition at 140 K which is attributed to a spin-density wave (SDW) and a tetragonal (*I*4/*mmm*) to orthorhombic (*Fmmm*) crystallographic structure transition. The superconductivity could eventually appear when the SDW transition is suppressed by applying physical pressure or chemical doping.

Compared with physical pressure, chemical doping has several alternative tuning modes, which can not only apply positive or negative chemical pressure but also introduce hole or electron carriers. For these reasons, it has been widely chosen as an effective method to investigate the superconductivity in BaFe_2_As_2_. For example, the superconductivity could be yielded by substituting isovalent P [6] or Ru [7] for As or Fe in order to introduce positive or negative chemical pressure, by substituting K for Ba to introduce hole carriers [3], and by substituting Co [8] or Ni [9] for Fe to introduce electron carriers. Unexpectedly, some other hole substitutions (Cr, Mn and V) at the Fe site could not induce superconductivity even though the SDW transition was also gradually suppressed [10,11,12]. The absence of hole carriers and a new competing *G*-type antiferromagnetic order has been proposed as a way to prevent the emergence of superconductivity in Mn- and Cr-doped BaFe_2_As_2_ [13,14]. However, even the strong hole-doping effect has been identified by Hall effect measurements in Ba(Fe_1−x_V_x_)_2_As_2_ [12], only local superconductivity along with coexisting magnetism is found to be consistent with the lack of bulk superconductivity [15]. This significant difference between hole doping and electron doping at the Fe site still provokes debate. Moreover, another much simpler correlation mechanism is proposed in a review article that suggests a decrease in *c*-lattice parameter is required to induce ‘in-plane’ (FeAs layer) superconductivity [16]. Based on this guideline, the bulk superconductivity induced by electron dopants at the Fe site and isovalent dopant at the As site with a smaller P element seems to be easily understood. Even for the isovalent Ru doping at the Fe site can be well interpreted, although the radius of the Ru ion is larger than the Fe ion, since it only expands lattice parameter *a* and the volume but indeed causes a decrease in lattice parameter *c* [7].

Recently, Jayalakshmi et al. theoretically proposed that the superconductivity is possible in (Ca,Sr,Ba)Fe_2_Bi_2_ compounds and BaFe_2_Bi_2_ might exhibit a higher *T*_C_ (≈30 K) than other proposed materials [17,18]. However, the structural data show that lattice parameter *c* is larger than BaFe_2_As_2_, which is contrary to the previous prediction that *c* should be reduced for ‘in-plane’ bulk superconductivity. Further work must be performed in order to verify whether BaFe_2_As_2_ could become a superconductor by using isovalent Bi as a substitution for As to introduce a negative pressure and whether the relationship between the *c* decrease and bulk superconductivity is still satisfactory. In this work, we report the synthesis and investigation of Bi-doped BaFe_2_As_2_ single crystals experimentally for the first time. Two filamentary superconducting phases with *T*_C_~25 K (SC I) and 15 K (SC III), and another possible bulk superconducting phase (SC II) with *T*_C_~7 K, are found, which indirectly confirms the possible superconductivity of BaFe_2_Bi_2_; however, the reduction in lattice parameter *c* for ‘in-plane’ bulk superconductivity might be not sufficient.

## 2. Materials and Methods

**Figure 1:**
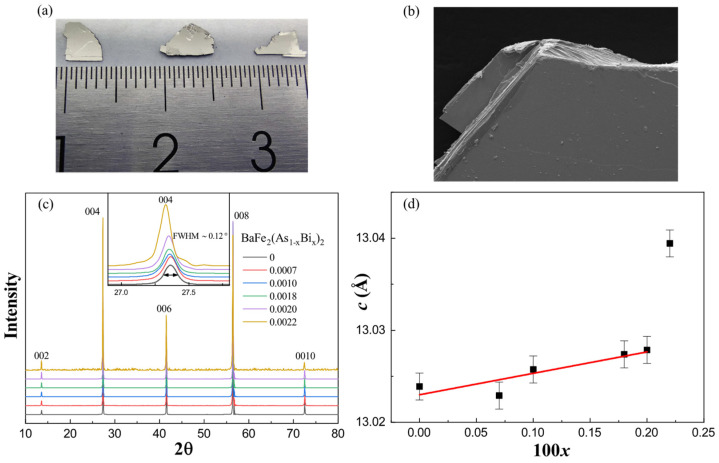
(**a**) The typical photograph, (**b**) the typical SEM surface morphology photograph, (**c**) the XRD patterns (inset is the enlarged (004) peaks and the FWHM is labeled) and (**d**) the concentration 100*x* dependence of lattice parameter *c* for BaFe_2_(As_1−x_Bi_x_)_2_ single crystals.

The BaFe_2_(As_1−x_Bi_x_)_2_ single crystals were synthesized by self-flux method. FeAs_1−x_Bi_x_ were prepared as precursors using the highly pure raw materials Fe, As and Bi by solid reaction method. The Fe, As and Bi powders were thoroughly mixed together and sealed in an evacuated quartz tube. The mixture was heated up to 750 °C and kept for 30 h. The obtained material was reground and sintered twice using the same heating procedure in order to make the Bi doping much more homogeneous. After we obtained the precursors, the Ba lump, as well as the FeAs and FeAs_1−x_Bi_x_ powders, were loaded in the alumina oxide tube and sealed in the evacuated quartz tube according to the stoichiometry ratio 1:2:2. The quartz tube was heated up to 1150 °C for 20 h and then slowly cooled down to 1000 °C with a rate of 2 °C/h in order to grow single crystals. In the end, the high-quality single crystals with dimensions up to 8 mm × 6 mm × 0.1 mm were obtained, as shown in Figure 1a. All the synthesis manipulations were carried out in a glove box filled with high-purity nitrogen gas.

The surface morphology and actual chemical compositions of BaFe_2_(As_1−x_Bi_x_)_2_ single crystals were examined by a scanning electron microscope (SEM) equipped with energy dispersive X-ray spectroscopy (EDX). The X-ray diffraction (XRD) was conducted with 2θ range from 10° to 80° on a Rigaku X-ray diffractometer (SmartLab SE, Tokyo, Japan) using Cu *K*_α_ radiation (λ = 1.5406 Å) generated at 40 kV and 30 mA. Both the resistivity and DC magnetic susceptibility was measured by the Quantum Design’s Magnetic Property Measurement System (MPMS3, San Diego, CA, USA). The resistivity was measured using the standard four-probe method, while the DC magnetic susceptibility was measured using the zero-field-cooling (ZFC) and field-cooling (FC) modes with two fields *H* = 20 Oe and 1 T along the *ab* plane.

## 3. Results and Discussion

### 3.1. Actual Doping Concentration and Lattice Parameter c

We have successfully grown five batches of BaFe_2_(As_1−x_Bi_x_)_2_ single crystals for the first time. The typical photograph of the single crystals in Figure 1a shows a quite shiny and flat surface, and the surface morphology in Figure 1b taken by SEM clearly displays its layered structure; both reflect the high quality of our single crystals. In order to determine the actual Bi doping concentration, we conducted the EDX measurement. The single crystals were first cleaved in order to present a shiny surface. Then, three different regions were chosen to detect the concentration for each sample. Since the Bi-doping level was quite low, it was not homogeneous, which is also compatible with our later-proposed scenario in which there may exist three different realms in our sample (R I, R II and R III). Therefore, we took the averaged concentration as the actual concentration. The nominal and actual Bi concentration *x* are listed in Table 1, and the actual doping concentration *x* will be used to label the single crystals hereafter. We can find that the actual concentration of Bi is quite a bit lower than the nominal value and, with the increase in the nominal doping level, the actual concentration first increases slightly, but further decreases when nominal *x* is above 0.2. The highest actual doping level could only reach up to 0.22%. This indicates that it is quite difficult to dope the Bi into the As site, which may be a result of the solid solubility limit or an unstable phase.

Figure 1c shows the XRD patterns of BaFe_2_(As_1−x_Bi_x_)_2_ single crystals. Only sharp (00*l*) peaks can be reflected on the patterns, suggesting that the cleaved planes are perpendicular to the *c*-axis. The inset is the enlarged (004) peaks. The high intensity and the narrow full width at half maximum (FWHM) labeled in the inset (2θ~0.12°) again indicate the high quality of our single crystals. Meanwhile, the (004) peak shifts to the lower diffraction angle with the increase in Bi concentration *x*, indicating that the Bi element is indeed introduced into BaFe_2_As_2_. The lattice parameter *c* for each doping sample was calculated using the XRD patterns and was plotted as a function of concentration 100*x* in Figure 1d. When *x* is below 0.002, the lattice parameter *c* increases almost linearly with the doping evolution, which is consistent with Vegard’s law [19], and it begin to deviate above 0.002 with a step increase in lattice parameter *c* for the *x* = 0.0022 sample. Compared with P dopant, Bi substitution for As expands the *c*-axis, presenting a negative pressure effect which also matches the expectation since the radius of the Bi ion is larger than that of the As ion.

### 3.2. Superconducting Transitions and Non-Fermi Liquid Behavior

**Figure 2:**
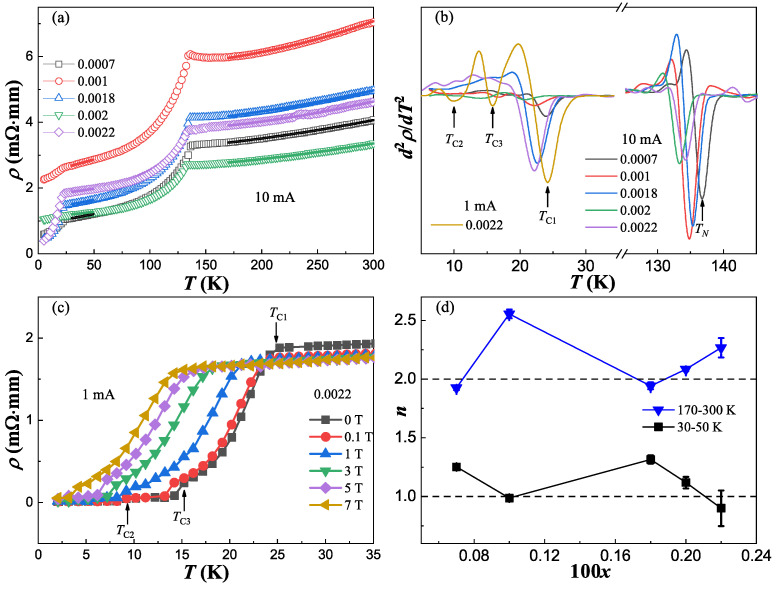
(**a**) The temperature dependence of resistivity measured with a 10 mA current for BaFe_2_(As_1−x_Bi_x_)_2_ single crystals. The solid lines are the fitting lines using the power law formula *ρ* = *ρ*_0_ + A*T^n^* between 30 K and 50 K, and between 170 K and 300 K. (**b**) The *d*^2^*ρ*/*dT*^2^ as a function of temperature according to the data from (**a**,**c**). (**c**) The temperature dependence of resistivity under different magnetic fields measured with a 1 mA current for the *x* = 0.0022 sample. (**d**) The concentration 100*x* dependence of fitting parameter *n* obtained from (**a**).

In order to investigate the superconducting properties, we first conducted resistivity measurements. Figure 2a displays the temperature dependence of resistivity down to 5 K measured with a 10 mA current for BaFe_2_(As_1−x_Bi_x_)_2_ single crystals. For all the samples, compared with the parent compound, a similar SDW transition around 135 K is found. But when temperature decreases to around 25 K, another transition happens with a drop of resistivity which may be associated with a superconducting transition. Actually, this kind of phenomenon is also observed in some BaFe_2_As_2_ [20,21], CaFe_2_As_2_ [22] and SrFe_2_As_2_ [23] parent compounds, as well as Ni-doped BaFe_2_As_2_ [24] and Pr-doped CaFe_2_As_2_ [25], which are proposed as filamentary superconducting transitions. For the *x* = 0.0022 sample, the percentage of resistivity dropping is highest: about 78%. Thus, we measured it again with lower 1 mA current and temperature down to 2 K, as shown in Figure 2c. Then, the zero resistivity was detected and, with the increase in the magnetic field, the transition temperature was suppressed gradually, confirming its superconducting properties. This significant dependence of resistivity on current is consistent with a more filamentary-natured superconductivity. To determine the antiferromagnetic (*T*_N_) and superconducting (*T*_C_) transitions, the temperature dependence of *d*^2^*ρ*/*dT*^2^ is plotted in Figure 2b and the minimum of *d*^2^*ρ*/*dT*^2^ is taken as the transition temperature marked by arrows. To our surprise, for the superconducting transition at low temperature, except for the one around 25 K (labeled as *T*_C1_), there seem to have been another two superconducting transitions around 15 K (labeled as *T*_C3_) and 10 K (labeled as *T*_C2_). These transitions are much clearer for the *x* = 0.0022 sample under 1 mA, which can also be identified easily by the naked eye, as marked by arrows in Figure 2c. Corresponding with the three temperatures, three superconducting phases are defined as SC I (*T*_C1_), SC II (*T*_C2_) and SC III (*T*_C3_).

The filamentary superconductivity (FL SC) has been argued to be attributed to the lattice distortion or strain [23], spin and orbital fluctuations [21], the antiphase domain walls [22] and the spontaneous electronic inhomogeneity at the nanoscale level [25]. Considering the average Bi concentration is quite low in our single crystals, we proposed our own scenario to interpret this behavior. We speculate that there might exist three kinds of realms in our sample: some realms (R I) are almost the same as the conditions found in the parent compound without any Bi dopants; some realms (R II) are highly Bi-doped; and the ratio of Bi doping in the rest of the realms (R III) is between the first two realms. The SC I with *T*_C1_, the SC II with *T*_C2_, and the SC III with *T*_C3_ originate from R I, R II and R III, respectively. The reasonable relationship between them will be discussed in detail later.

In iron pnictides, the normal state of resistivity has been extensively described by the power law formula *ρ* = *ρ*_0_ + A*T^n^*, where *n* is the temperature exponent, *ρ*_0_ is the residual resistivity, and A is a constant. Here, to avoid the antiferromagnetic (AFM) and superconducting transitions’ influence, the 30–50 K (above *T*_C1_ and below *T*_N_) and 170–300 K (above *T*_N_) ranges are chosen as two fitting regions. The solid lines shown in Figure 2a represent the perfect fitting results using the power law. The evolution of *n* with Bi doping is summarized in Figure 2d. There is no significant doping dependence of *n* for both regions. However, *n* is much closer to 2 above *T*_N_, thus corresponding to a Fermi-liquid (FL) ground state, while *n* is closer to 1 below *T*_N_ before the emergence of superconductivity, thus corresponding to a non-Fermi-liquid (NFL) behavior. This anomalous NFL behavior has often been revealed just above the superconducting dome, which may be closely tied to superconductivity [26,27]. Moreover, a simultaneous disappearance of the superconductivity and the NFL transport is observed in CaFe_2_(As_1−x_P_x_)_2_ [28]. From our results, this explicit indication of a FL to NFL crossover from normal state to AFM order state confirms the idea that NFL behavior may play a crucial role in the presence of superconductivity.

### 3.3. Superconducting Volume and Effective Moment

**Figure 3:**
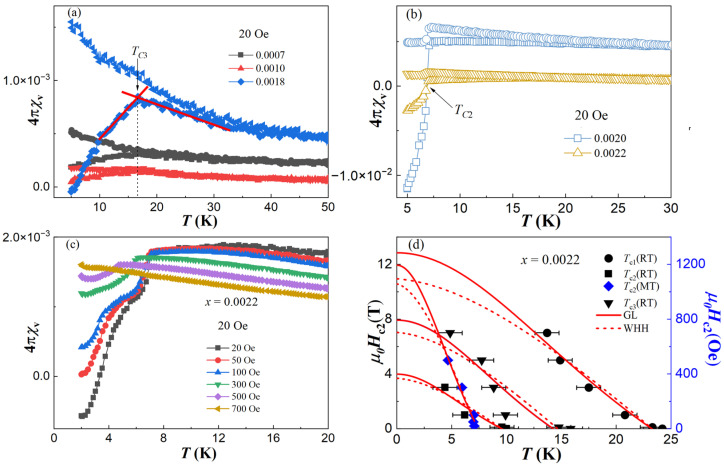
The temperature dependence of magnetic susceptibility for (**a**) BaFe_2_(As_1−x_Bi_x_)_2_ (*x* = 0.0007, 0.0010 and 0.0018) with *H* = 20 Oe magnetic field, (**b**) *x* = 0.0020 and 0.0022 with *H* = 20 Oe magnetic field, and (**c**) *x* = 0.0022 under different magnetic fields. (**d**) The temperature dependence of *µ*_0_*H*_c2_ for *x* = 0.0022 sample. The black points corresponding to the left coordinate axis are obtained from resistivity data in Figure 2c, and the blue points corresponding to the right coordinate axis are from the magnetization data in (**c**). The red solid lines are the fitting lines according to GL function, and the red dash lines are according to WHH function.

To further investigate the superconducting diamagnetism, the temperature dependence of magnetic susceptibility was measured using an *H* = 20 Oe magnetic field by the zero-field-cooling (ZFC) and field-cooling (FC) modes, as shown in Figure 3a–c. In contrast with the three superconducting transitions observed in resistivity, the one at *T*_C1_ could no longer be detected, while the one at *T*_C3_ was only detected in the low Bi-doping single crystals with *x* = 0.0007, 0.0010 and 0.0018 at 17 K, as shown in Figure 3a; the one at *T*_C2_ was only detected in the high Bi-doping single crystals with *x* = 0.0020 and 0.0022 at 7 K, as shown in Figure 3b. The absence of SC I in the magnetic susceptibility confirms its filamentary-natured superconductivity. Although the SC III is present, the superconducting transition is broad and the shielding signal magnitude is very weak, so that only the *x* = 0.0018 sample shows a diamagnetic property below 6 K, indicating a very small superconducting volume fraction. The absolute superconducting volume (SC *V*) is calculated by 4π*χ*_v_(*T*_C_) − 4π*χ*_v_(5 K), as shown in Figure 5a (left axis). The SC *V* for *x* = 0.0007, 0.0010 and 0.0018 is about 0.01%, 0.01% and 0.09%, respectively. This indicates that the SC III is not a bulk superconductor, but it does reflect that the superconductivity is less filamentary compared with SC I, and the SC *V* increases with the increase in Bi doping. Especially with the further increase in Bi concentration, a very sharp superconducting transition and a dramatically enhanced shielding signal are observed for SC II, as shown in Figure 3b. The SC *V* for *x* = 0.0020 and 0.0022 has increased significantly: up to about 1.65% and 0.34%. With the increase in Bi doping, the SC *V* changes by order of magnitude, crossing from 0 for SC I to 10^−2^ order for SC III and then to 10^−1^ or 1 order for SC II. Since the chemical substitution can create local, hence average, structural changes which would then greatly impact the electronic structure, the lattice effect of Bi doping could not be ignored. This suggests that these three superconducting phases and doping levels are mutually related. Therefore, the scenario we proposed previously regarding the relationship between the three superconducting phases (SC I, SC II and SC III) and the three realms (R I, R II and R III) has been shown to be reasonable and easily understood. This can also be reflected by the disappearance of the SC I and SC II for *x* = 0.0007, 0.0010 and 0.0018, as shown in Figure 3a. Although both of them are invisible, the reasons are different. For SC I, it is the result of its filamentary nature’s superconductivity property, which is related to the realm with almost no Bi doping, but for SC II, it is highly related to the Bi-doping level and its disappearance is attributed to the lack of Bi concentration. Moreover, compared with the SC *V* for *x* = 0.0007, the relative variation ratio (*V* − *V*(*x* = 0.0007))/*V*(*x* = 0.0007) is also plotted as a function of 100*x*, as shown in Figure 5a (right axis). Even though all of our single crystals show a non-bulk superconductivity with SC *V*, less than 2%, the relative variation ratio has a dramatic increase, up to 600%, 13,300% and 2565% for *x* = 0.0018, 0.0020 and 0.0022, respectively, clearly heralding the emergence of a possible bulk superconductivity (Bulk SC) for SC II. The reason why the bulk superconductivity was not shown in our sample is that the actual Bi concentration is still too low. We wonder if a true bulk superconductor could have emerged if the Bi concentration had been further increased. This idea is also supported by the theoretical calculation that the parent BaFe_2_Bi_2_ might hold *T*_C_~30 K superconductivity [18].

To study the upper critical field *µ*_0_*H*_C2_, the magnetic susceptibility for *x* = 0.0022 was measured again, down to 2 K, under various magnetic fields, up to 700 Oe, as shown in Figure 3c. As the field increases, the superconducting transition is gradually suppressed to a lower temperature, confirming its superconductivity properties. Combined with the resistivity and magnetic susceptibility, by taking the data from Figure 2c and Figure 3c, the *T*_C_ value at each field is plotted as *µ*_0_*H*_C2_ versus *T*, as shown in Figure 3d. The dashed curves are fits to the Werthamer–Helfand–Hohenberg (WHH) relation [29], *H*_C2_(0) = −0.693*T*_C_*(dH*_C2_/*dT*)_*T*=*Tc*_, and the solid curves are fits to Ginzburg–Landau (GL) model: *H*_C2_(*T*) = *H*_C2_(0) [(1 − *t*^2^)/(1 + *t*^2^)], where t represents the normalized temperature *T*/*T*_C_. The obtained fitting parameters of *T*_C_ and *µ*_0_*H*_C2_ are summarized in Table 2. The values of *T*_C_ are consistent with each other while the values of *µ*_0_*H*_C2_(0) obtained from WHH are lower than GL, which is similar to those reported in other iron pnictide superconductors [30,31]. These values are much smaller than the nominal Pauli paramagnetic limit, which is roughly estimated by *µ*_0_*H^P^* = 1.84*T*_C_ [32], indicating the orbital limit effect. The GL coherence length *ξ_GL_*, shown in Table 2, is also calculated using the relation *µ*_0_*H*_C2_(0) = *Φ*_0_/(2π$\xi_{GL}^{2}$), where *Φ*_0_ = 2.07 × 10^−15^ T·m^2^ is the flux quantum.

**Figure 4:**
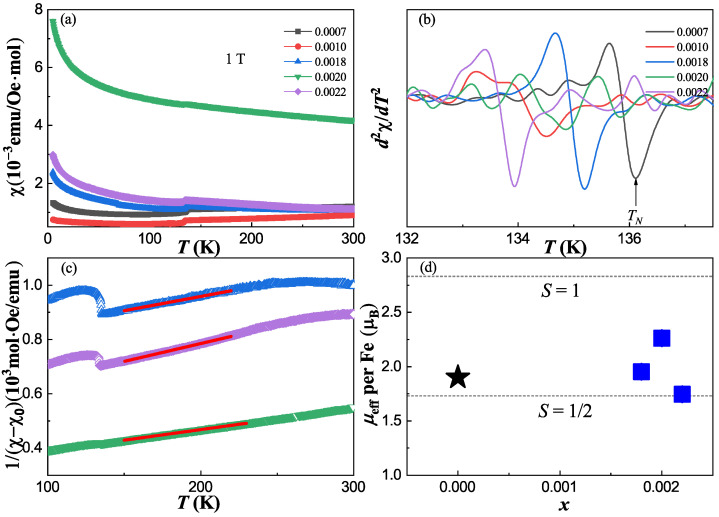
(**a**) The temperature dependence of magnetic susceptibility for BaFe_2_(As_1−x_Bi_x_)_2_ single crystals with *H* = 1 T magnetic field. (**b**) The *d*^2^*χ*/*dT*^2^ as a function of temperature according to the data from (**a**). (**c**) The temperature dependence of 1/(*χ* − *χ_0_*) for *x* = 0.0018, 0.0020 and 0.0022 samples. The red solid lines are fits to the Curie–Weiss law. (**d**) The effective moment per Fe site as a function of the doping concentration *x*. The blue square points are our results, and the black star point is from reference [34].

Magnetic susceptibility is also measured by applying the *H* = 1 T magnetic field, as shown in Figure 4a. The SDW transitions are observed for all the single crystals and the minimum of *d*^2^*χ*/*dT*^2^ plotted in Figure 4b is taken as the AFM transition temperature, which is consistent with the resistivity measurement. Above *T*_N_, the magnetic susceptibility shows a *T*-linear behavior for *x* = 0.0007 and 0.0010, which is universal in iron pnictides and is explained by the existence of a wide antiferromagnetic fluctuation [33]. When *x* > 0.0018, the deviation from *T*-linear behavior becomes obvious and gradually changes to a Curie–Weiss-like behavior. As shown in Figure 4c, the magnetic susceptibilities can be well fitted between 150 K and 220 K by the Curie–Weiss law 1/(χ − χ_0_) = (*T* − *θ*_CW_)/*C*, where χ_0_ is the temperature-independent magnetic susceptibility, *θ*_CW_ is the Curie temperature and *C* is the Curie–Weiss constant. The extracted effective moment *μ*_eff_ per Fe is plotted in Figure 4d. The values are about 1.95*μ*_B_, 2.26*μ*_B_ and 1.74*μ*_B_ for *x* = 0.0018, 0.0020 and 0.0022, corresponding to an effective spin close to *S* = ½, and comparable with a value of about 1.9*μ*_B_ for the parent compound BaFe_2_As_2_, as studied by inelastic neutron scattering [34]. It seems that the effective moment is almost independent from the Bi substitution. In contrast, the effective moment gradually decreases with increasing *x* for the other two isovalent substitution systems in BaFe_2_(As_1−x_P_x_)_2_ [35,36] and Ba(Fe_1−x_Ru_x_)_2_As_2_ [37,38]. Especially for the Ba(Fe_1−x_Ru_x_)_2_As_2_ system, Ru substitution also presents a negative pressure effect, but with a decrease in the *c*-axis. The dilution of the magnetic moment at the Fe site is contributed to a decrease in *z*_As_, which is correlated with a decrease in the *c*-axis [38]. Therefore, the doping independence of effective moment in our BaFe_2_(As_1−x_Bi_x_)_2_ sample may be linked to the increase in the *c*-axis.

### 3.4. Superconducting Phase Diagram

**Figure 5:**
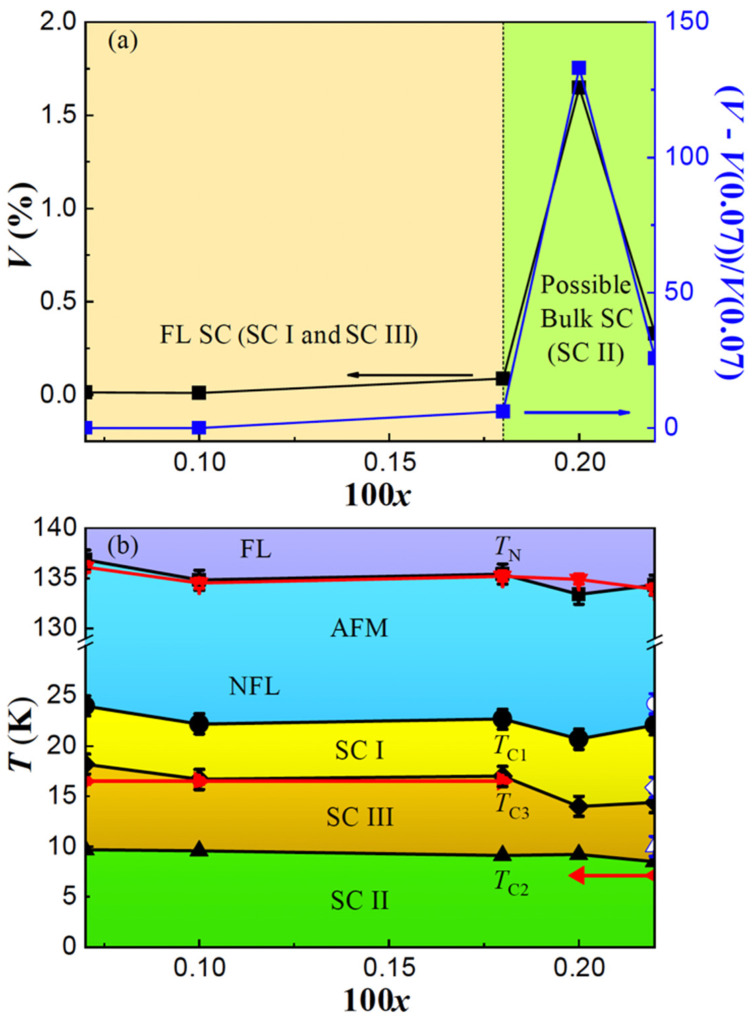
(**a**) The concentration 100*x* dependence of superconducting volume (left axis) and the relative variation ratio (*V* − *V*(*x* = 0.0007))/*V*(*x* = 0.0007) (right axis). (**b**) The phase diagram of BaFe_2_(As_1−x_Bi_x_)_2_ as a function of concentration 100*x*. The solid black (empty blue) points are derived from resistivity measured with 10 mA (1 mA) current. The solid red points are derived from magnetic susceptibility.

Based on the resistivity and magnetic susceptibility data, we constructed a complete temperature-doping phase diagram of BaFe_2_(As_1−x_Bi_x_)_2_ which is presented in Figure 5b. One AFM phase and three superconducting phases labeled as SC I, SC II and SC III are well defined and coexistent. The *T*_C_ is about 25 K, 15 K and 7 K for SC I, SC III and SC II, respectively. Attributed to the relatively low Bi concentration, the coexistence of the AFM state and superconductivity might only occur in the microscopic region. In addition, both the AFM and superconducting transition temperatures seem to be independent from the slight Bi variation. From the normal state to the AFM order state, a FL to NFL crossover is revealed and the NFL behavior appears right at the boundary of superconducting phases, which may be a significant factor driving the presence of superconductivity. The red data points in the superconducting region demonstrate that only the SC II and SC III could be detected by the diamagnetic signal. Combined with the dramatic enhancement of SC *V* in Figure 5a, we strongly suggest that these superconducting phases are closely correlated with the Bi concentration; the SC I and SC III should be filamentary superconductors, while the SC II should be a bulk superconductor.

## 4. Conclusions

In conclusion, a series of high-quality BaFe_2_(As_1−x_Bi_x_)_2_ single crystals were successfully grown for the first time. The highest doping level could only reach up to 0.22%, and the Bi doping enlarged lattice parameter *c*, showing a negative pressure effect. By investigating resistivity and magnetic susceptibility, a FL to NFL crossover from normal state to AFM state, and three superconducting phases labeled as SC I, SC II and SC III, were observed. We assume that the NFL behavior plays a crucial role in the presence of superconductivity. The evolution of SC *V* with Bi doping suggests that these superconducting phases should be highly related to the Bi doping concentration, and the SC II with *T*_C_~7 K is proposed as a possible bulk superconductor. Thus, the previous prediction regarding the superconductivity in parent BaFe_2_Bi_2_ is possible, and the reduction in lattice parameter *c* for ‘in-plane’ bulk superconductivity might be not sufficient. To further confirm our scenarios regarding the origin of the three superconducting phases, we recommend that scanning tunneling microscopy measurements be conducted for specific investigation in the future.

## Figures and Tables

**Table 1:** The nominal and actual doping concentration of BaFe_2_(As_1−x_Bi_x_)_2_ single crystals.

Nominal *x*	Actual *x*
0.01	0.0007
0.05	0.0020
0.10	0.0018
0.20	0.0022
0.35	0.0010

**Table 2:** The superconducting transition temperature *T*_C_, upper critical field *µ*_0_*H*_C2_ and GL coherence length *ξ_GL_* parameters obtained from WHH and GL fits for the three superconducting phases derived from resistivity and magnetic susceptibility measurements for the *x* = 0.0022 sample.

*x* = 0.0022	WHH Fit		GL Fit		
	*T*_C_ (K)	*µ*_0_*H*_C2_ (T)	*T*_C_ (K)	*µ*_0_*H*_C2_ (T)	*ξ_GL_* (Å)
SC I (RT)	23.3	10.91	23.2	12.90	51
SC II (RT)	9.6	3.67	9.6	3.98	91
SC II (MT)	7.3	0.11	7.4	0.12	524
SC III (RT)	14.7	7.01	14.3	7.93	65

## Data Availability

Data are available upon request to the corresponding author.
